# Supplementary figures and images for: A comparison of non-surgical methods for sexing young gopher tortoises (Gopherus polyphemus)
Source: PeerJ. 2022 Jun 14;10:e13599. doi: 10.7717/peerj.13599 (PMC9205304; doi:10.7717/peerj.13599)

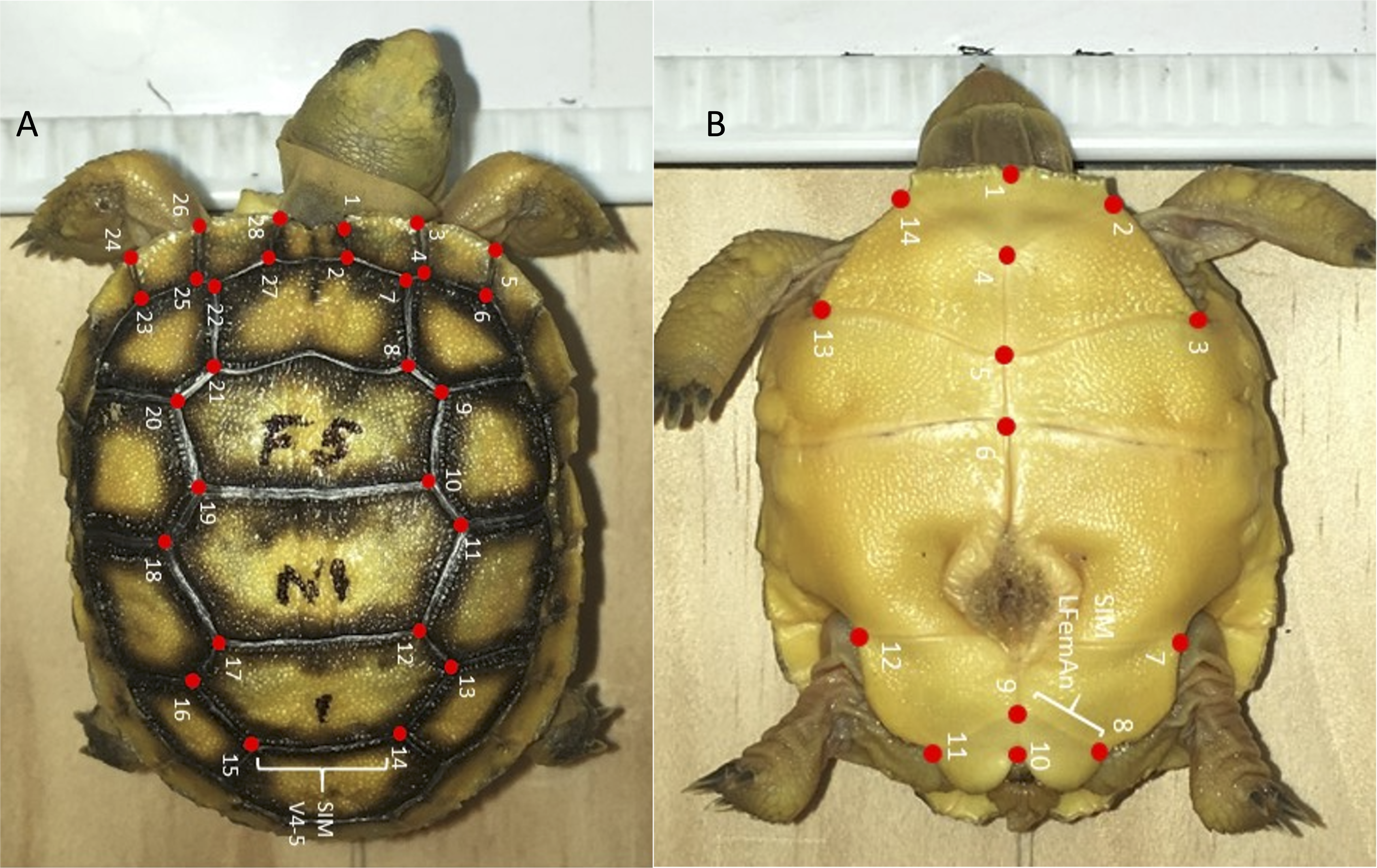

Supplement: Figure S1 — We used tpsDIG to place landmarks on standardized photos of each individual’s (A) top of the carapace (top) and (B) plastron (bottom). Photo credit: Kevin Loope [file peerj-10-13599-s003.png]

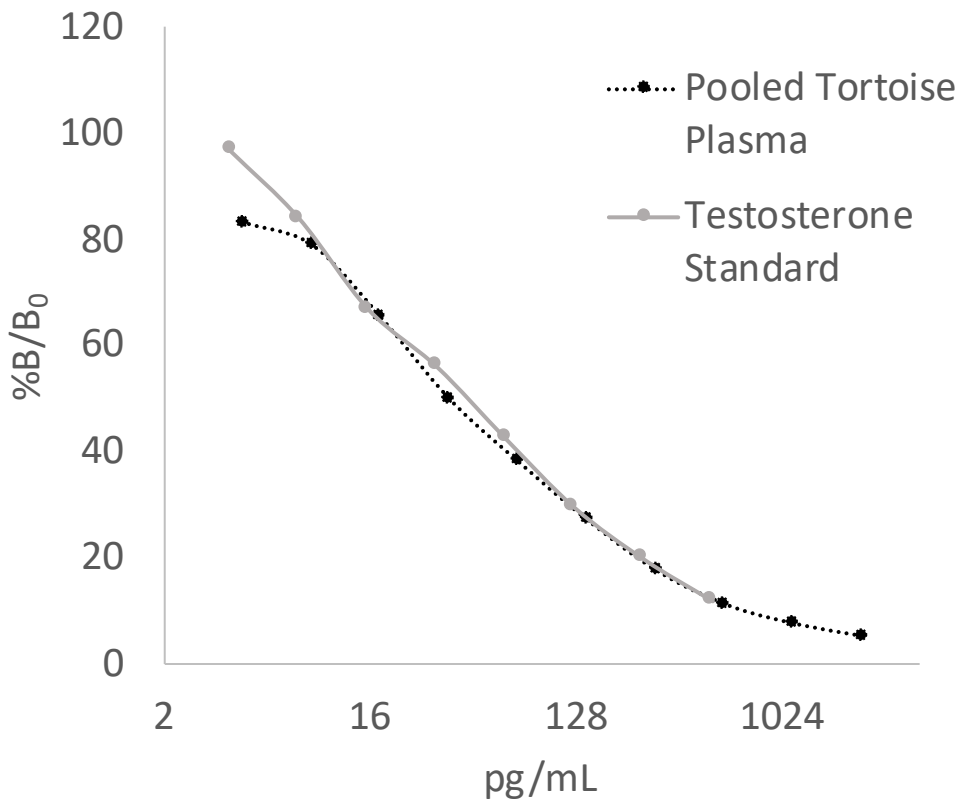

Supplement: Figure S2 — The two-fold serial dilution of a pool of tortoise plasma (1 to 1/1024; n = 20 adult females) is parallel to a two-fold serial dilution of the testosterone standard (Cayman Biological: 500 pg/uL to 3.9 pg/ul). This parallelism suggests that the ELISA antibodies are targeting the same antigens in each series, and thus that the kit is detecting testosterone in plasma samples. It also suggests a lack of matrix interference by other compounds in the plasma. %B/B_0 = %bound/maximum bound [file peerj-10-13599-s004.pdf]

# Saline injections

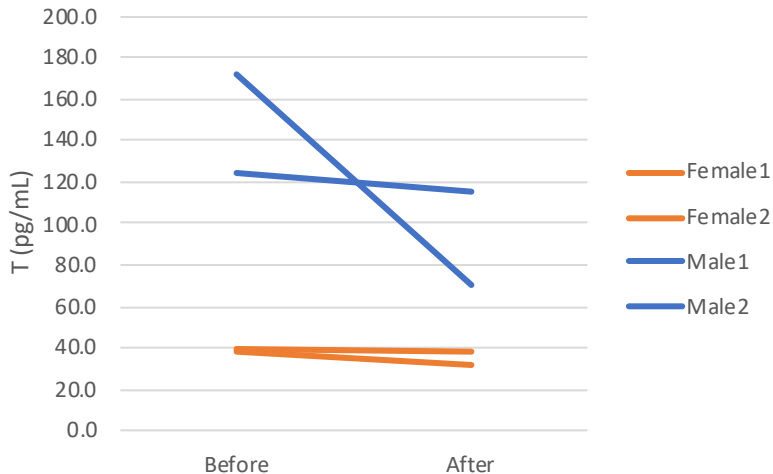

Supplement: Figure S3 [file peerj-10-13599-s005.pdf]
