# Supplementary material for: A comparison of non-surgical methods for sexing young gopher tortoises (Gopherus polyphemus)
Source: PeerJ. 2022 Jun 14;10:e13599. doi: 10.7717/peerj.13599 (PMC9205304; doi:10.7717/peerj.13599)
Supplement: Table S1 [file peerj-10-13599-s006.docx]

Table S1. Importance values for testosterone and morphological measurements in the neonate post-FSH testosterone + morphology random forest model, ordered by mean decrease in accuracy.

|  | female | male | MeanDecreaseAccuracy | MeanDecreaseGini |
| --- | --- | --- | --- | --- |
| concentration | 0.155 | 0.024 | 0.053 | 3.671 |
| cw.sh | 0.091 | 0.028 | 0.042 | 2.529 |
| pmax | 0.056 | 0.02 | 0.028 | 1.278 |
| sh | 0.043 | 0.005 | 0.014 | 1.73 |
| cw.v | 0.018 | 0.009 | 0.011 | 0.694 |
| vol | 0.016 | 0.009 | 0.01 | 0.659 |
| pmax.v | 0.031 | 0.004 | 0.01 | 1.174 |
| cw | 0.019 | 0.008 | 0.01 | 0.657 |
| pmin.v | 0.014 | 0.009 | 0.01 | 0.692 |
| pmin | 0.016 | 0.007 | 0.009 | 0.746 |
| sh.v | 0.01 | 0.007 | 0.008 | 0.527 |
| mcl.v | 0.013 | 0.005 | 0.007 | 0.571 |
| cw.mcl | 0.008 | 0.005 | 0.006 | 0.648 |
| mcl | 0.006 | 0.005 | 0.005 | 0.425 |
